# Supplementary material for: Imaging Based Techniques Combined with Color Measurements for the Enhancement of Medieval Wall Paintings in the Framework of EHEM Project
Source: J Imaging. 2024 Jun 29;10(7):159. doi: 10.3390/jimaging10070159 (PMC11278335; doi:10.3390/jimaging10070159)
Supplement: Supplementary file 1 [file jimaging-10-00159-s001.zip › jimaging-3034122-supplementary.pdf]

Article

Imaging based techniques combined with color measurements for the enhancement of Medieval wall paintings in the framework of EHEM project

Paola Pogliani [1], Claudia Pelosi [2]*, Luca Lanteri[2] and Giulia Bordi [4]

[1] DIBAF Department, University of Tuscia, Largo dell'Università, 01100 Viterbo, Italy
[2] DEIM Department, University of Tuscia, Largo dell'Università, 01100 Viterbo, Italy
[3] Department of Humanities, University of Rome 3, Via Ostiense 234, 00146 Roma, Italy
* Correspondence: pelosi@unitus.it; Tel.: +39-0761-357673

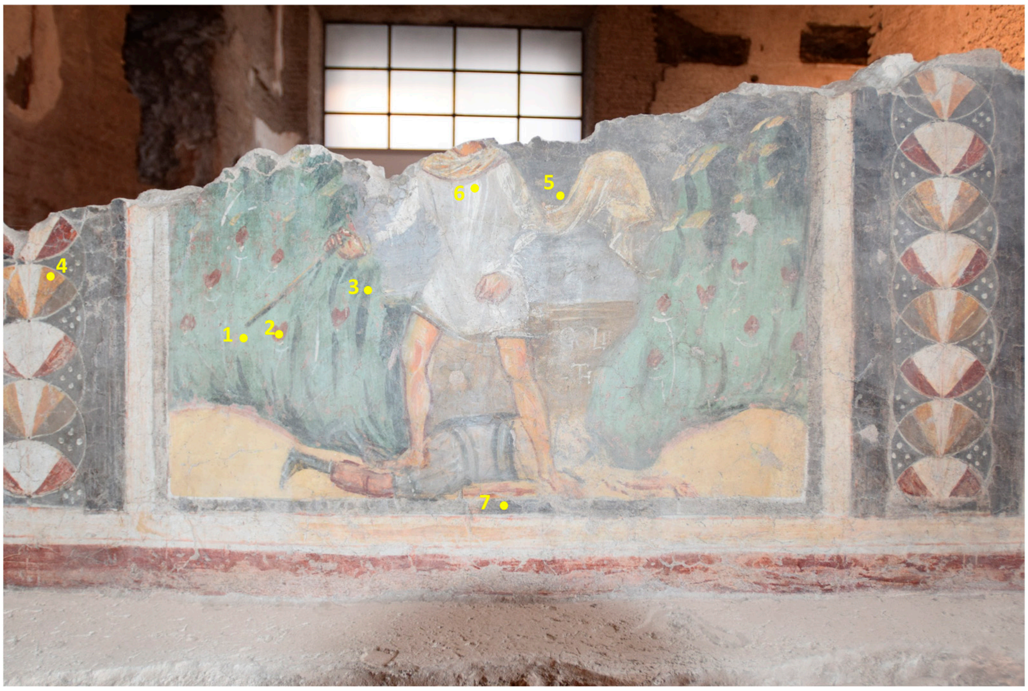

Figure S1. Areas 01 (high choir, west wall) with the points of color spot measurements.

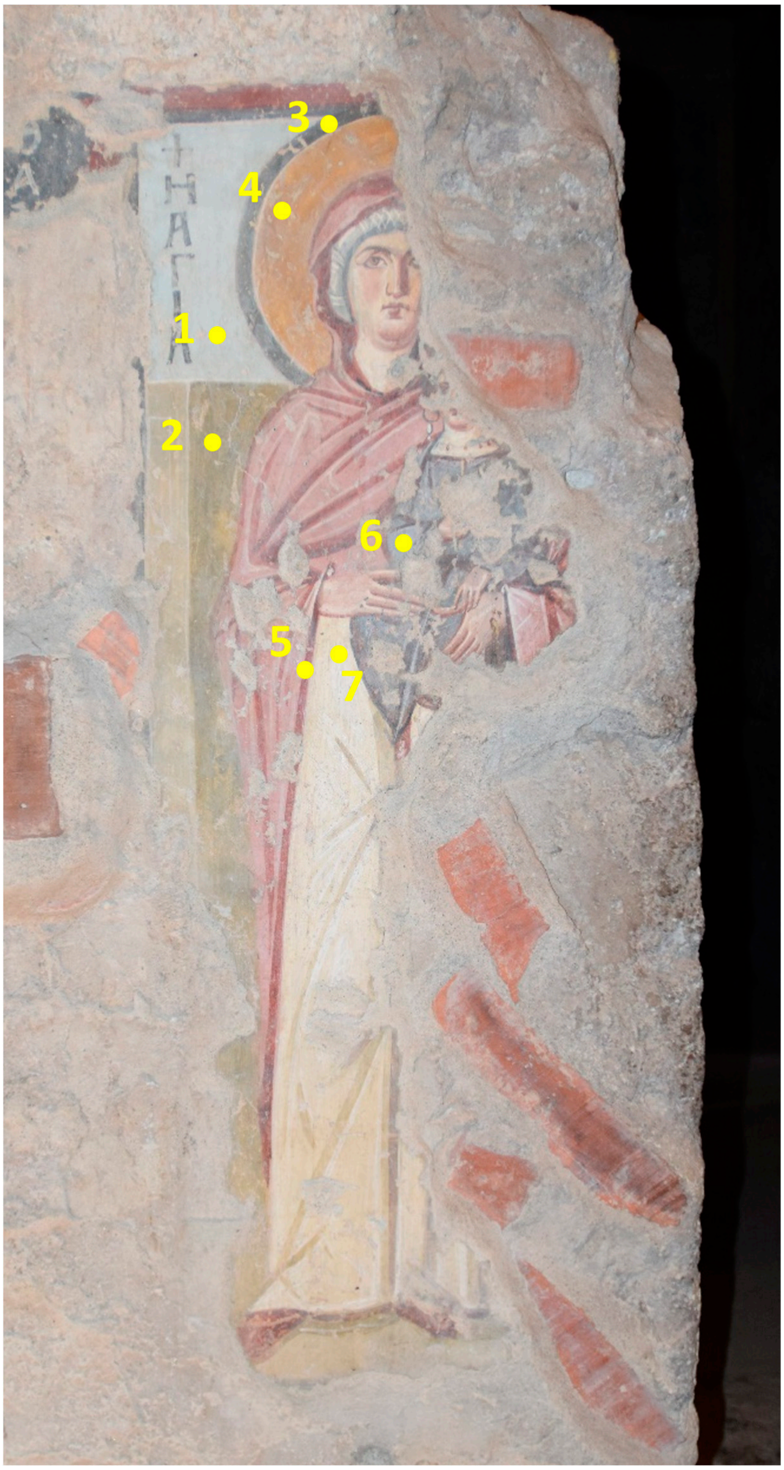

Figure S2. Areas 02 (Saint Anne and Child Mary) with the points of color spot measurements.

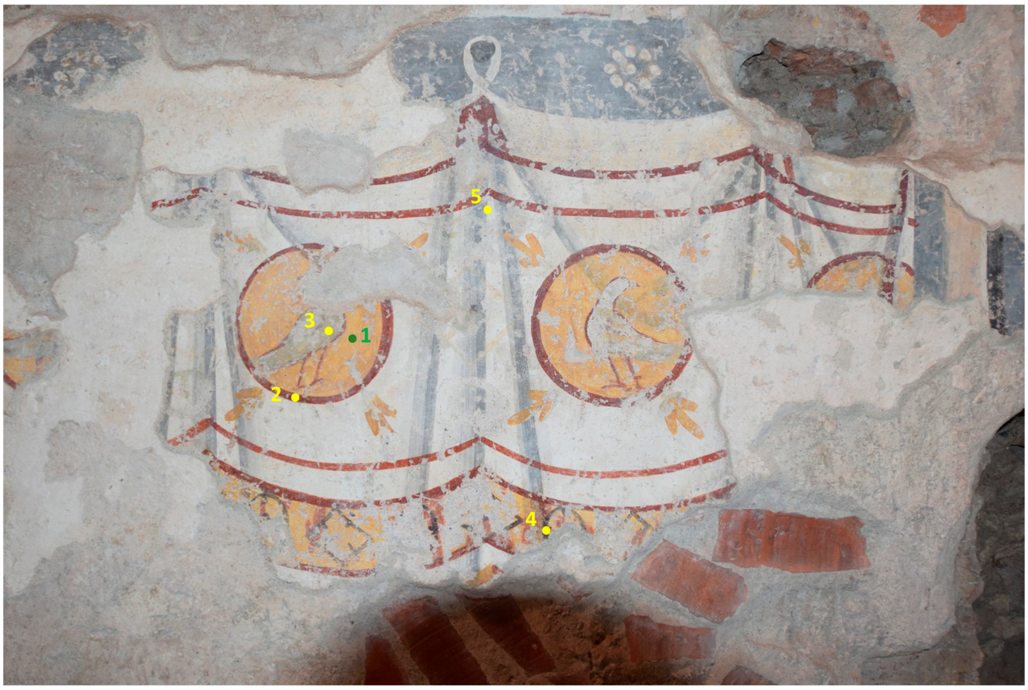

Figure S3. Areas 03 (left wall in respect to the apse) with the points of color spot measurements.

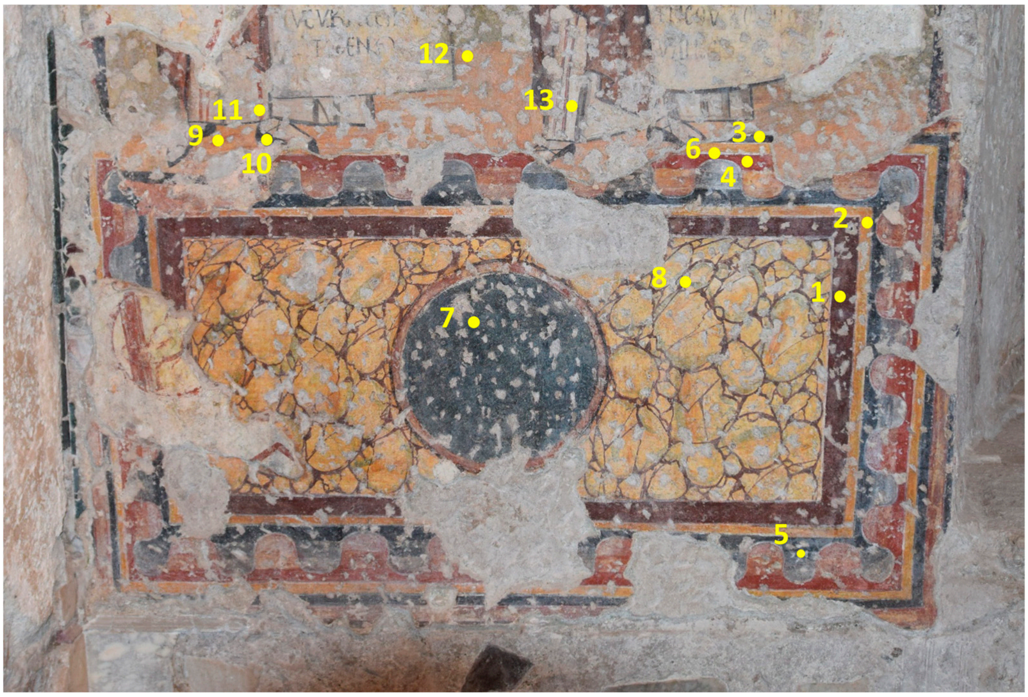

Figure S4. Areas 04A (lower side, false marble decoration, points 1-8) and areas 04B (upper side the 663 AD pictorial phase, points 9-13) with the points of color spot measurements.

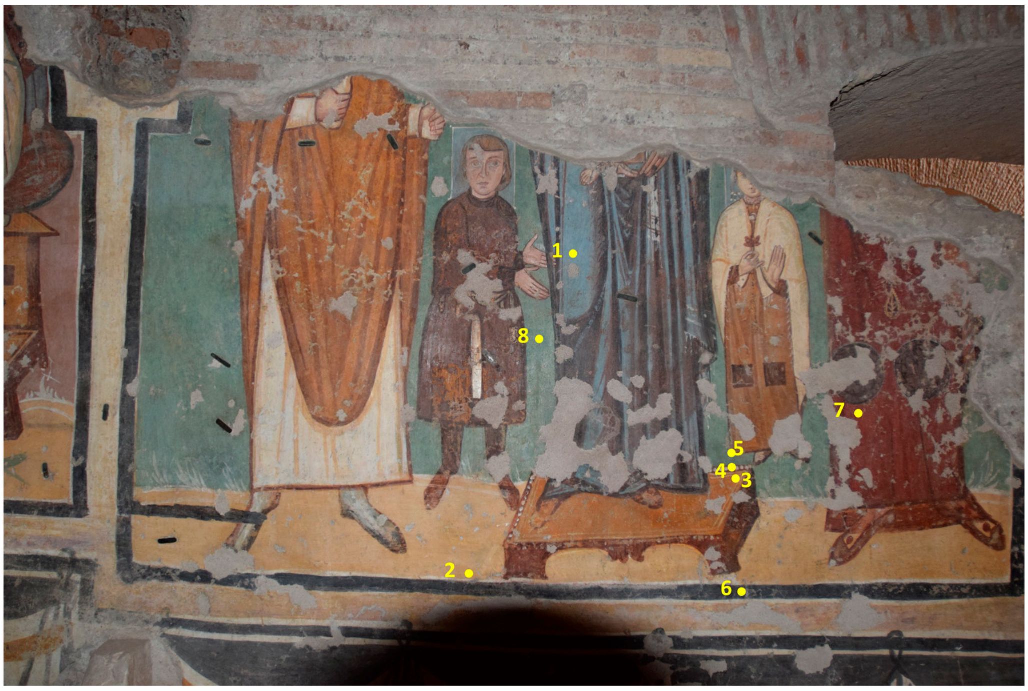

Figure S5. Areas 05 (Theodotus chapel) with the points of color spot measurements.

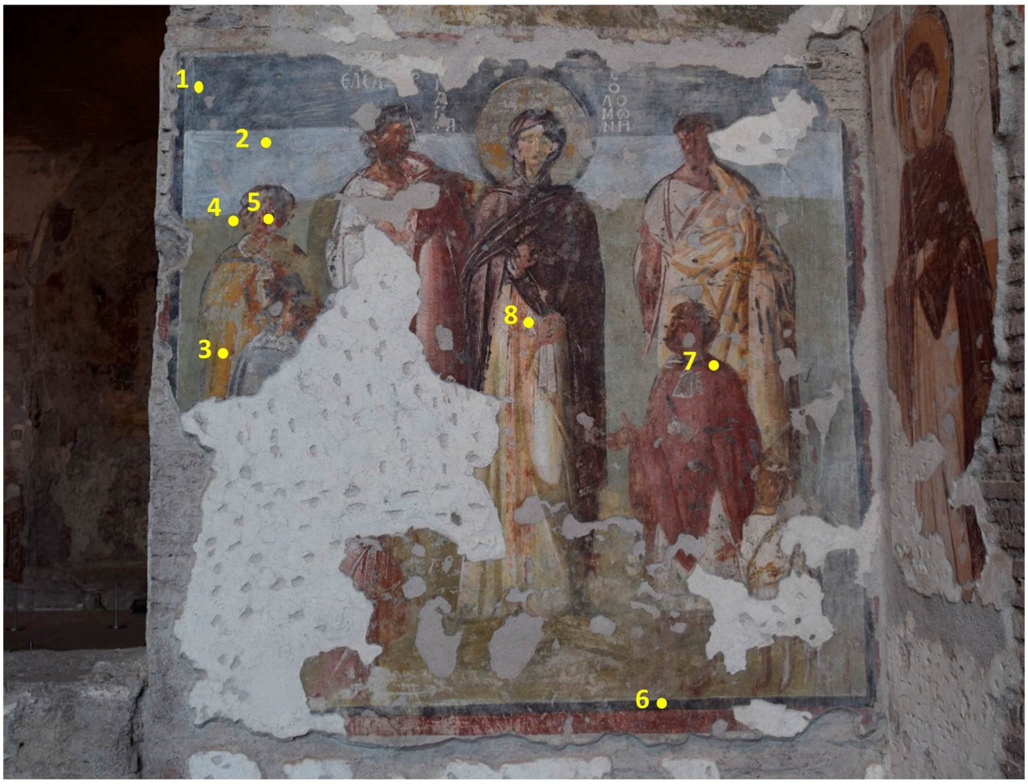

Figure S6. Areas 06 representing Solomon and the Maccabees with the points of color spot measurements.

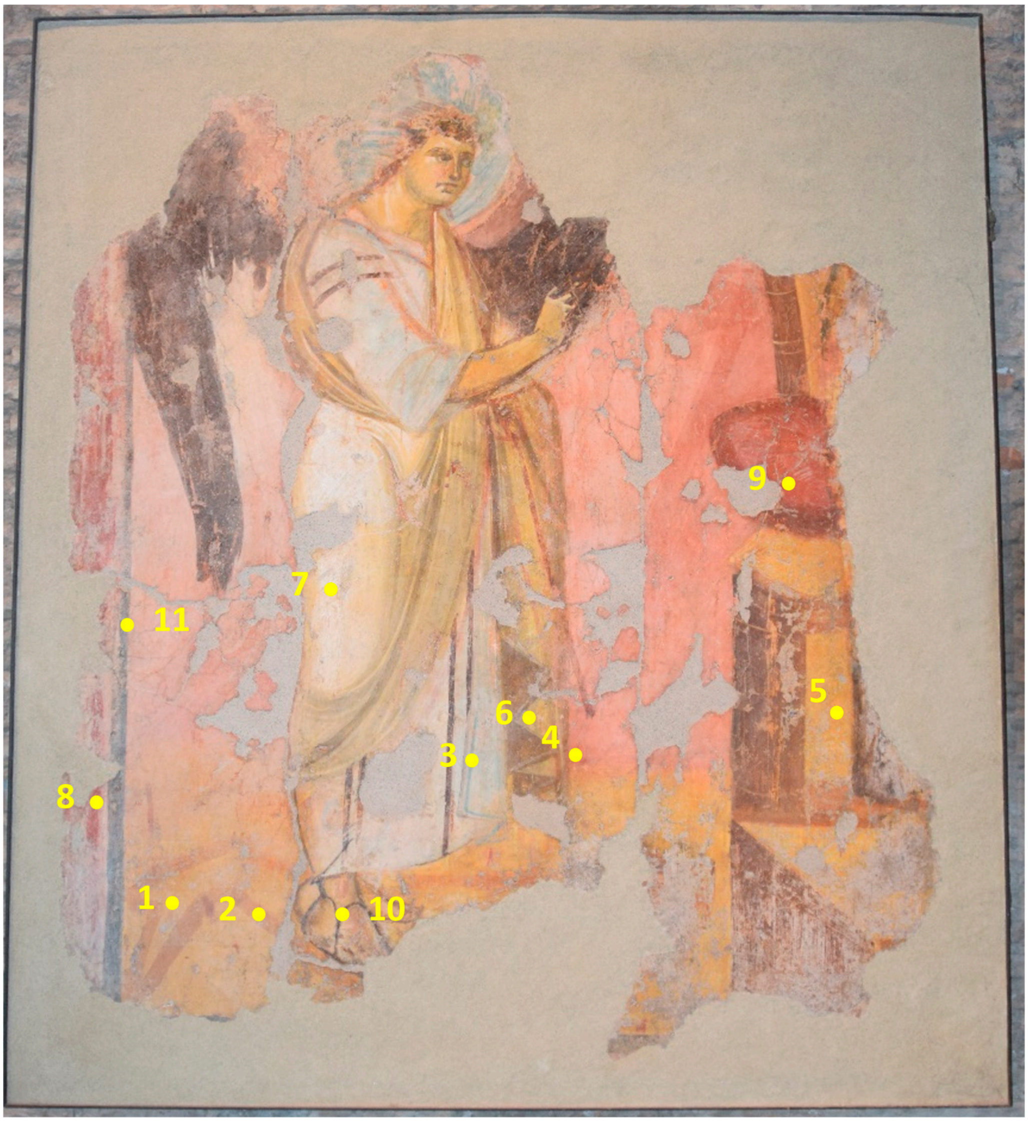

Figure S7. Areas 07 showing the Annunciation (pictorial phase of the Pope John) with the points of color spot measurements.

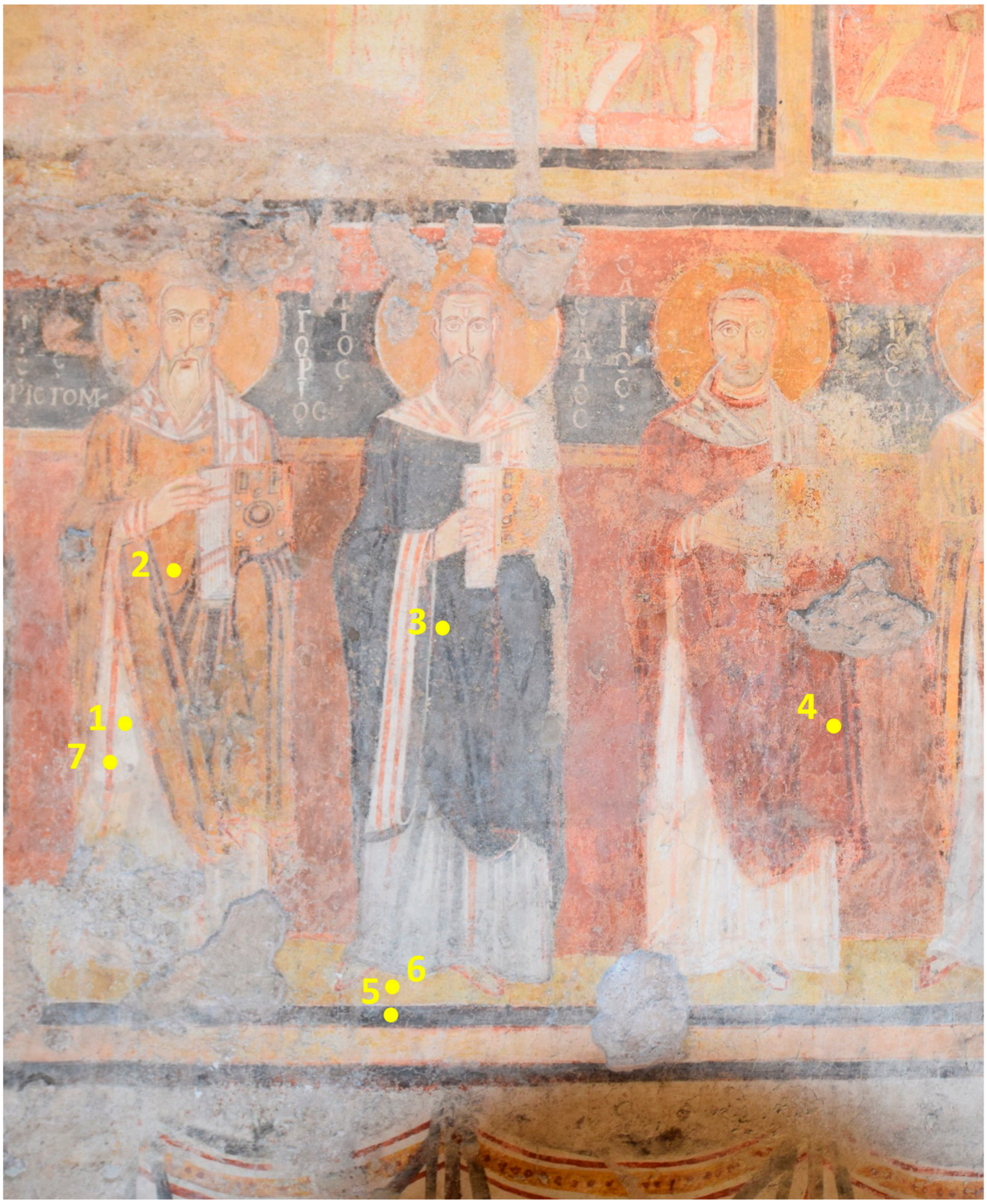

Figure S8. Areas 08 attributed to the Pope Stephen II, with the points of color spot measurements.
